# Ectopic Spacer Acquisition in *Streptococcus thermophilus* CRISPR3 Array

**DOI:** 10.3390/microorganisms9030512

**Published:** 2021-03-01

**Authors:** Rodrigo Achigar, Martina Scarrone, Geneviève M. Rousseau, Cécile Philippe, Felipe Machado, Valentina Duvós, María Pía Campot, Moïra B. Dion, Yuyu Shao, María Julia Pianzzola, Sylvain Moineau

**Affiliations:** 1 Laboratorio de Biotecnología, Facultad de Ingeniería, Universidad ORT Uruguay, Montevideo 11100, Uruguay; rodrigoachigar@gmail.com (R.A.); felipe@enteria.uy (F.M.); valentina@enteria.uy (V.D.); pia@enteria.uy (M.P.C.); 2 Département de Biochimie, De Microbiologie et de Bio-Informatique, Faculté des Sciences et de Génie, Université Laval, Québec, QC G1V 0A6, Canada; martina.scarrone96@gmail.com (M.S.); genevieve.rousseau@greb.ulaval.ca (G.M.R.); cecilemphilippe@gmail.com (C.P.); moira.dion.1@ulaval.ca (M.B.D.); yuyu.shao.1@ulaval.ca (Y.S.); 3 Groupe de Recherche en Écologie Buccale, Faculté de Médecine Dentaire, Université Laval, Québec, QC G1V 0A6, Canada; 4 Departamento de Biociencias (DEPBIO), Facultad de Química, Universidad de la República, Montevideo 11800, Uruguay; mpianzzo@gmail.com; 5 Félix d’Hérelle Reference Center for Bacterial Viruses, Faculté de Médecine Dentaire, Université Laval, Québec, QC G1V 0A6, Canada

**Keywords:** *Streptococcus thermophilus*, *Streptococcus pyogenes*, CRISPR-Cas, adaptation, phages

## Abstract

*Streptococcus thermophilus* relies heavily on two type II-A CRISPR-Cas systems, CRISPR1 and CRISPR3, to resist siphophage infections. One hallmark of these systems is the integration of a new spacer at the 5′ end of the CRISPR arrays following phage infection. However, we have previously shown that ectopic acquisition of spacers can occur within the CRISPR1 array. Here, we present evidence of the acquisition of new spacers within the array of CRISPR3 of *S. thermophilus*. The analysis of randomly selected bacteriophage-insensitive mutants of the strain Uy01 obtained after phage infection, as well as the comparison with other *S. thermophilus* strains with similar CRISPR3 content, showed that a specific spacer within the array could be responsible for misguiding the adaptation complex. These results also indicate that while the vast majority of new spacers are added at the 5′ end of the CRISPR array, ectopic spacer acquisition is a common feature of both CRISPR1 and CRISPR3 systems in *S. thermophilus*, and it can still provide phage resistance. Ectopic spacer acquisition also appears to have occurred naturally in some strains of *Streptococcus pyogenes*, suggesting that it is a general phenomenon, at least in type II-A systems.

## 1. Introduction

*Streptococcus thermophilus* is a lactic acid bacterium used extensively for the manufacture of several fermented dairy products, such as yogurt and several cheeses [1,2,3,4]. Siphophage infections of these Gram-positive bacteria are the leading cause of milk fermentation failures worldwide [5,6,7,8]. One important strategy for controlling virulent phages in industrial settings is to select and use natural bacteriophage-insensitive mutant (BIM) strains as starter cultures. It is now well-documented that *S. thermophilus* strains rely on the CRISPR-Cas system (a prokaryotic adaptive immune system) to protect itself against phage attacks [9,10,11,12,13,14,15]. This system is composed of a clustered regularly interspaced short palindromic repeats (CRISPR) array and its associated *cas* genes [16,17,18]. These *cas* genes encode Cas proteins, some of which are used by the bacteria to acquire new immunities by integrating short DNA sequences, called spacers, from invading DNA, such as phage genomes, at the leader 5′ end of the CRISPR array. Then, the CRISPR array is transcribed and processed into small interfering RNAs (called crRNAs) [19,20]. These crRNAs form ribonucleoprotein complexes with Cas proteins and destroy invading DNA through base-pair recognition and cleavage [21,22,23].

CRISPR-Cas systems are currently divided into two classes, six types, and several subtypes [24]. For most of them, the integration of new spacers is mostly driven by two proteins, Cas1 and Cas2, which form an integrase-like complex [25]. During the adaptation process, the first repeat of the CRISPR array is also duplicated upstream of the newly acquired spacer [26]. *S. thermophilus* strains have been shown to possess up to four different CRISPR-Cas systems: two distinct type II-A systems, one type I-E, and one type III-A [22]. So far, only the two type II-A systems, CRISPR1 (CR1) and CRISPR3 (CR3), appear to be active in spacer acquisition [27,28], with the large majority of the acquisition events taking place in the CR1 array [29], which is the most predominant system in this bacterial species.

Type II-A CRISPR-Cas systems are composed of four genes coding for Cas9, Cas1, Cas2, and Csn2 [30]. All four are required for the adaptation step in vivo, which implies probable interactions between them [31]. For type II CRISPR-Cas systems, the signature gene is *cas9*, which encodes a multidomain protein that combines the functions of the crRNA–effector complex, targets DNA cleavage, and contributes to the selection of new spacers during the adaptation stage [30]. Cas9 recognizes short motifs called protospacer adjacent motifs (PAMs) within the invading DNA. When a PAM is recognized, the adjacent protospacer sequence can be integrated into the CRISPR array as a new spacer. Csn2 also appears to be critical in the adaptation stage as a *csn2* gene insertion mutant was found to be incapable of acquiring new spacers in response to phage infection [3,11]. Of note, the two type II-A systems of *S. thermophilus* use a different PAM [21,22,27].

Cryoelectron microscopy analyses of the Cas1-Cas2-Csn2 complex from the *S. thermophilus* CR3 system showed a large multi-subunit complex (Cas1_8_-Cas2_4_-Csn2_8_) with a channel occupied by (approx. 30 bp) double-stranded DNA, also suggesting a protective role for the complex. Spacer adaptation complexes may have quite different architectures, but the speculated model for spacer capture suggest that: (1) the Cas1-Cas2-Csn2 complex engages free DNA ends from invading dsDNA phage genome and encircles it within the complex, (2) the Cas1-Cas2-Csn2 complex slide on the DNA until it encounters Cas9 that is bound to a PAM, (3) DNA is cleaved, releasing Cas9 and the Cas1-Cas2-Csn2 complex, encapsulating the DNA as a new spacer ready for integration [31].

One of the hallmarks of the acquisition of new immunities is that novel spacers are typically integrated at the leader-proximal region of the CRISPR array [32]. This polarity is guided by the leader sequence upstream of the CRISPR array [29]. It is likely that sequences within the leader elements of CRISPR loci are important given that novel spacers are introduced adjacent to the leader in several systems. It was reported that the integrity of the 3’ end of the leader sequence (called leader anchoring sequence or LAS) is crucial for the polarized acquisition of new spacers [33]. It has been also shown that Cas1 contains a DNA-binding region that binds this leader DNA [34,35,36]. This sequence (most notably the 5′-GAG-3′ at the 3′ end) is highly conserved across type II systems. In the absence of an appropriate LAS, other short nucleotide sequences within the *Streptococcus pyogenes* type II-A associated CRISPR array were shown to guide ectopic (acquired at positions other than the 5′ end) spacer integration in the heterologous host *Staphylococcus aureus* [33]. Ectopic spacer acquisition was also recently observed in the CRISPR array associated with the type II-A system of *Streptococcus mutans* [37].

In a previous study, we observed ectopic spacer acquisition in the CR1 array of *S. thermophilus* BIMs, obtained after a phage-sensitive host was exposed to virulent phages of the *Siphoviridae* family [38]. In this study, we investigated whether ectopic spacer acquisition could also occur following a phage infection in the second active type II-A CRISPR-Cas system of *S. thermophilus*, namely CR3, as well as determining if the presence of a LAS sequence was needed.

## 2. Materials and Methods

### 2.1. Phage and Bacterial Strains

Virulent siphophage 53 (*cos*-type) was previously isolated from a failed mozzarella production in Uruguay and its complete genome is available (accession no. KT717084) [38]. Phage-sensitive *S. thermophilus* strains (Uy series) were obtained from a local starter culture supplier and grown in LM17 medium at 42 °C. Phages and bacterial strains were stored, as frozen stocks, in LM17 supplemented with 15% (v/v) glycerol. Phage 53 was amplified in LM17 supplemented with 10 mM CaCl_2_ (LM17-CaCl_2_). Briefly, 0.1 mL of a fresh bacterial culture (OD_600_ = 0.6) was inoculated in 10 mL of broth and incubated at 42 °C for three hours. Then, 0.1 mL of phage lysate was added and incubated at 42 °C until complete lysis was observed. The lysate was filtered (0.45 µm filters) and stored at 4 °C until used. Phage titer was determined using methods described elsewhere [39].

### 2.2. S. thermophilus and S. pyogenes CRISPR Loci Analysis

The CR3 loci of 50 strains of the Uy collection were amplified by PCR (NEB Phusion High-Fidelity DNA Polymerase) and sequenced using primers CR3-fwd (5-CTGAGATTAATAGTGCGATTACG-3) and CR3-rev (5-GCTGGATATTCGTATAACATGTC-3) [27]. Bioinformatics analyses to identify the CR3 spacer content of 45 Uy strains were firstly performed with SnapGene (version 4.1.9). In addition, 39 CR3 loci were retrieved from 64 complete *S. thermophilus* genomes available in GenBank as of October 2020. Similarly, type II-A CRISPR loci were searched from *S. pyogenes* genomes. A set of 213 complete and circular genomes was available from GenBank as of October 2020. All type II-A loci (116 for *S. pyogenes* and 84 for *S. thermophilus*) were identified with CRISPRDetect (version 2.2.3, http://crispr.otago.ac.nz/CRISPRDetect, accessed on 1 March 2021) using default parameters [40]. The putative CRISPRs were also manually checked. CRISPRDetect gff output files were then used with the Python script CRISPRStudio (version 1) [41] to extract, align and cluster the spacer sequences to generate a SVG file for CRISPR loci representation. Output SVG files were edited manually using Illustrator 2020 or Inkscape 1.0.0.

### 2.3. Bacteriophage Insensitive Mutants (BIMs)

BIMs were obtained by infecting the phage-sensitive strain *S. thermophilus* Uy01 with the virulent phage 53. Briefly, approximately 5 × 10^8^ CFU of *S. thermophilus* were mixed with 1 × 10^8^ PFU of phages in 4 mL of soft LM17-CaCl_2_ (0.75% agar) and poured on a LM17-CaCl_2_ agar plate (1.5% agar). Plates were incubated at least 48 h at 42 °C. Individual colonies were recovered, streaked and re-streaked for purity. Individual colonies were then inoculated and incubated overnight in LM17 broth. The cultures were then tested for phage resistance as described elsewhere [10].

## 3. Results

### 3.1. Evidence of Ectopic Acquisition Events in CRISPR3

**Figure 1:**
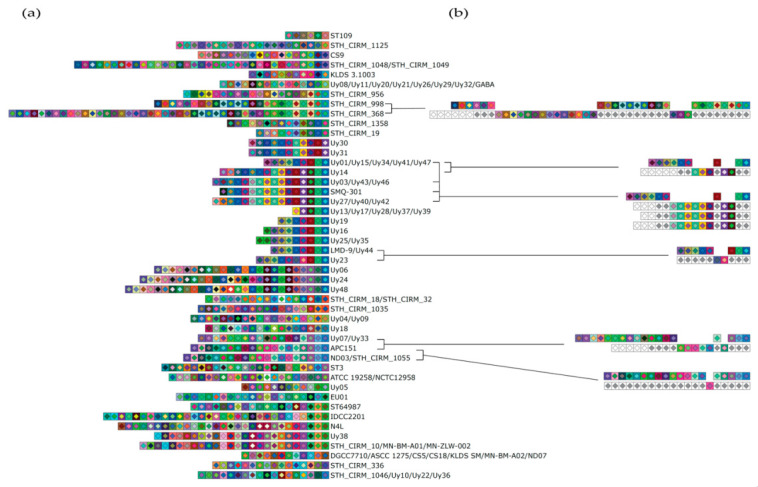
Spacer representation of CRISPR3 loci of *S. thermophilus* genomes using CRISPRStudio. (**a**) CRISPR3 loci of complete public *S. thermophilus* genomes and of strains of the Uy collection. (**b**) Selected examples of probable ectopic spacer acquisition or insertion/deletion events in CRISPR3 loci. The order of the spacers is the same as in panel a. White square harboring a light gray diamond represents similar spacer. White square harboring a white diamond represents unique spacer on the 5′ end of the array.

The analysis of the spacer content of the CR3 array was performed on 64 complete public *S. thermophilus* genomes available at the time of the study in GenBank along with 50 strains of the Uy collection. A total of 39 (60.9%) CR3 loci were retrieved from publicly available *S. thermophilus* genomes and 45 (90%) CR3 loci were detected by PCR from the Uy stain collection. Analysis of these arrays showed that they contained a minimum of 5 spacers and a maximum of 44, with a median of 15 spacers per strain. Moreover, the same spacers appeared to have been acquired by some of these wild-type strains or were derived from a common ancestor (Figure 1a). These data also suggested that spacers were either deleted or acquired at specific positions within the CR3 array (Figure 1b). For example, when comparing *S. thermophilus* strain Uy23 with the strain Uy44 and the reference strains LMD-9 and Uy44, it would appear that two spacers were either deleted in the CR3 array of the Uy44/LMD-9 strains or were acquired by the strain Uy23. Similar events of either deleted or ectopically acquired spacers appear to have occurred within the CR3 arrays of other strains (Figure 1b). In some strains, new spacers appear to have also been subsequently acquired at the 5′ end of the array, as with the strain APC151 when compared to strains Uy07 and Uy33.

We next sought to identify similar potential and natural ectopic acquisition events in other type II-A systems, outside *S. thermophilus*. The type II-A system of *S. pyogenes* system has been shown to acquire spacer ectopically when expressed in a heterologous host [33] Thus, we explored if natural ectopic spacer acquisition could also be inferred from publicly available genomic sequences of *S. pyogenes*. Moreover, the Cas9 (1368 aa) of *S. pyogenes* is related to *S. thermophilus* CR3 Cas9 (1409 aa, 57% ID). Interestingly, the *S. pyogenes* system uses the PAM 5′-NGG-3′, while the CR3 system of *S. thermophilus* uses the PAM 5′-NGGNG-3′ [22,27].

From the 213 *S. pyogenes* genomes found in GenBank, 116 (54.5%) CRISPR arrays with at least three repeats were detected. Analysis of these arrays showed that they contained up to a maximum of 13 spacers, with a median of 4 spacers per strain. Of note, five strains (2.3%) with only two repeats and one spacer were also detected. Therefore, the number of spacers is generally smaller in *S. pyogenes* than in *S. thermophilus*.

**Figure 2:**
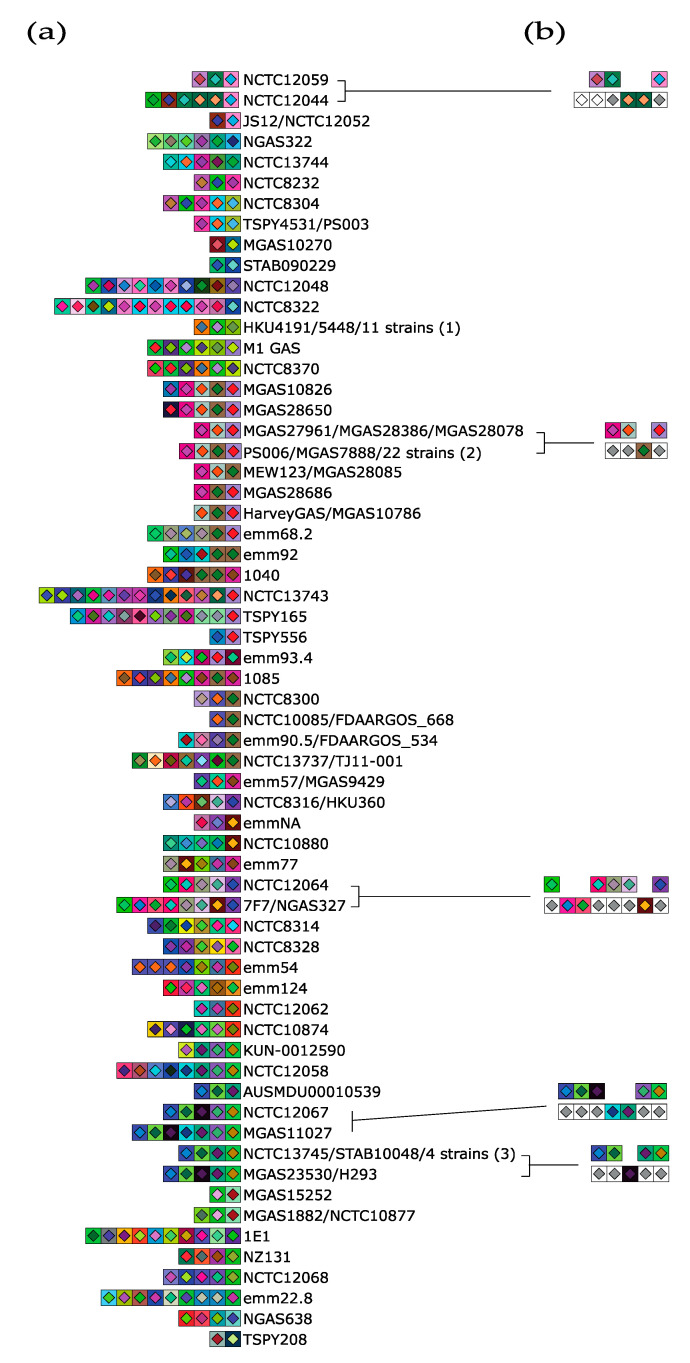
Spacer representation of CRISPR type II-A loci of *S. pyogenes* genomes using CRISPRStudio. (**a**) Type II-A CRISPR loci from public *S. pyogenes* genomes and containing at least two spacers. Some strains have identical CRISPR arrays and it includes Group 1: Strains S119, MGAS2221, emm1, FDAARGOS_149, HKU488, 5448, 10-85, MTB314, M1 476, MGAS5005, A20. Group 2: MGAS7914, MGAS8347, MGAS11052 MGAS11108, MGAS11115, MGAS28191 MGAS28271, MGAS28278, MGAS28330, MGAS28360, MGAS28533, MGAS28669, MGAS28746, MGAS29064, MGAS29284, MGAS29326, MGAS29409, M28PF1, STAB09014, STAB10015, MGAS6180, NIH35. Group 3: STAB09023, MGAS27061, JMUB1235, KUN-0014944. (**b**) Examples of possible ectopic acquisition or insertion/deletion events in *S. pyogenes* CRISPR arrays. The order of the spacers is the same as in panel a. White square harboring a light gray diamond represents similar spacer. White square harboring a white diamond represents unique spacer on the 5′ end of the array.

Further analyses suggested that some of the strains acquired the same spacers or that the strains were derived from a common ancestor (Figure 2a). Moreover, specific spacers could have also been either deleted or acquired within the CRISPR array (Figure 2b). For example, when comparing *S. pyogenes* strain NCTC12044 with the strain NCTC12059, two spacers appear to have been deleted in the CRISPR array of strain NCTC12059 or acquired by the strain NCTC12044. The latter strain may also have acquired two unique spacers at the 5′ end, compared to the reference strain.

### 3.2. Evaluation of Spacer Acquisition

**Figure 3:**
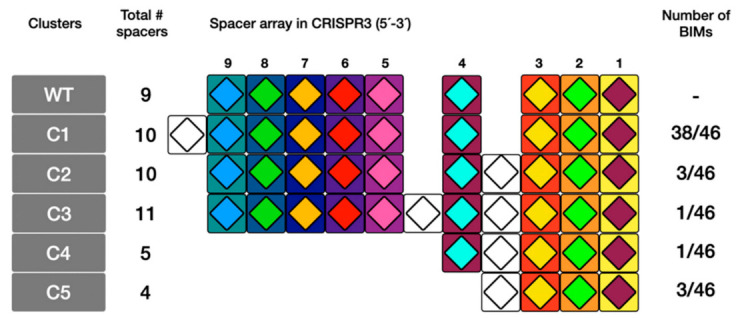
Ectopic spacer acquisition events observed for Uy01 bacteriophage insensitive mutants (BIMs). Spacers are aligned and their direction is shown from 5′ to 3′, with respect to the leader sequence. The position of each spacer is relative to the leader end of the reference wild-type (WT) strain Uy01, with spacer 1 being the most distal. Each spacer is represented by a combination of colors based on their respective nucleotide sequence. The white spacers indicate that various spacers were acquired at these positions. The letter C followed by a number indicates a BIM cluster.

**Figure 4:**
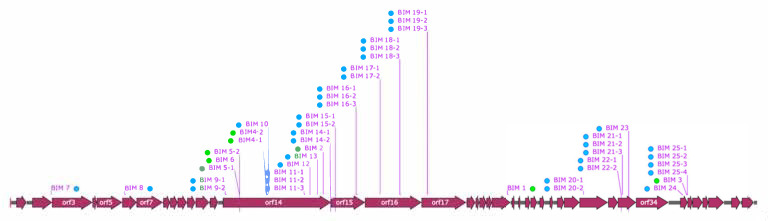
Graphic representation of phage 53 genome (accession no. KT717084). The arrows indicate the different open reading frames. The blue dots show the position of the protospacers acquired at the 5′ end of the CR3 array by the BIMs obtained in this work. The green dots represent the position of protospacers acquired ectopically. The BIM number represents the different protospacers, and a hyphen followed by a number indicates that the protospacer was incorporated by more than one BIM.

To test if ectopic acquisition can indeed occur in the CR3 array of *S. thermophilus*, bacteriophage insensitive mutants (BIMs) were generated by infecting the phage-sensitive *S. thermophilus* strain Uy01 (which has nine spacers in its CR3 array) with the virulent phage 53 for a prolonged period of time. Forty-six randomly selected BIMs were analyzed for new spacer acquisition. All of them acquired new spacers in the CR3 array. Only one spacer was acquired per BIM, except for BIM1, which acquired two. A total of 25 different spacers (out of 47) were acquired by these 46 BIMs (Supplementary Table S1), indicating that some BIMs acquired the same spacer. We observed clear ectopic spacer acquisition events in the CR3 of 5 BIMs (10.9%), representing three CRISPR cluster (C) types (C2, C3 and C4; Figure 3). Spacer acquisitions with concomitant spacer deletions were also observed in four BIMs (8.7%) (see C4 and C5, Figure 3). It is unclear if additional ectopic spacer acquisitions also occurred in the three BIMs grouped in C5 (Figure 3). The remaining 38 BIMs acquired spacers at the leader 5′ end of the CRISPR array (CR1, Figure 3). Interestingly, over a third (16) of all the acquisition events involved protospacers from *orf14* (the longest gene in the phage genome which codes for the tape measure protein), including six of the eight ectopic events (Figure 4). All the protospacers were flanked by the previously identified PAM for CR3 (5′-NGGNG-3′) [4,8].

Most ectopic acquisition events occurred between spacers 3 and 4. A single ectopic spacer acquisition also occurred between spacers 4 and 5 in BIM1. Interestingly, loss of spacer content also occurred in three of these BIMs, namely BIMs 3, 4, and 6 (Figure 3). The newly acquired spacers were mapped in the phage genome and perfectly matched protospacers (Table 1), except for one new spacer that could not be matched to the phage genome. The BIMs were tested against phage 53 and all showed a phage-resistant phenotype, including those that had acquired the spacer in the middle of the array.

**Figure 5:**
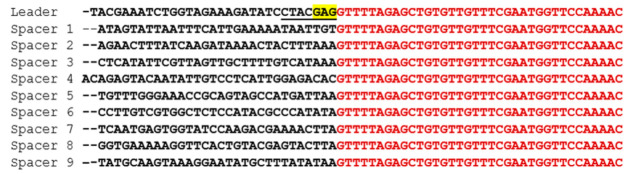
Nucleotide alignment of the leader sequence and spacers of *S. thermophilus* Uy01. The nucleotide sequence of the repeat is in red and the leader and spacers are in black. The underlined portion of the sequence is the putative LAS sequence of the *S. thermophilus* CRISPR3 array with the GAG motif highlighted in yellow.

Because the region flanking spacers 3 and 4 appears to be a hot spot for ectopic spacer acquisition in CR3, we investigated the presence of a motif that could mimic the leader sequence, as previously observed in ectopic events in CR1 of *S. thermophilus* [38]. In both cases, spacers did not contain the GAG motif found in the LAS sequence in the leader sequence (Figure 5). Only the adenine at position -2 matching the GAG motif was found in five spacers, including spacers 3 and 4.

## 4. Discussion

Integration of new spacers at the 5′ end of the CRISPR loci is one of the hallmark features of the CRISPR-Cas systems in *S. thermophilus* [42] and other bacterial species [3], although ectopic spacer acquisition in CR1 was previously described in *S. thermophilus* [38]. Our results now show that ectopic spacer acquisition can also occur in the CR3 system of *S. thermophilus*. Spacer analysis of CRISPR type II-A loci of *S. pyogenes* genomes hinted at ectopic spacer acquisition in this species as well. BIMs were generated by infecting the phage-sensitive *S. thermophilus* strain Uy01 with the virulent phage 53. All of them acquired spacers in CR3. We also observed that many of the newly acquired spacers came from protospacers found in the *orf14* (coding for the tape measure protein) of phage 53, without any obvious reason, other than being the longest gene. Of interest, there are 478 CR3 PAMs in the genome of phage 53, and 68 of them (14%) are in *orf14*. The absence of spacer acquisition in the CR1 of this strain is unknown, but it could be related to the presence of genes coding for anti-CRISPR proteins in the phage or bacterial genome [43,44,45] or a defect in the spacer acquisition machinery.

Recent work showed the importance of a seven-nucleotide sequence at the 3′ end of the leader sequence, called the “leader-anchoring sequence” (LAS), in the acquisition of new spacer [42]. When this (or similar) LAS sequence is found in specific spacers, this may lead to ectopic spacer acquisition events, especially if they have a GAG motif at the 3′ end [16]. In a previous study, we observed that ectopic integration occurred when the last G of the leader sequence was missing, and that new spacers could be added at five different positions within the array [38]. We noticed that in four out of the five adjacent spacers to the newly acquired one, an adenine and a guanine were found at position -2 and -1 (3′ end), respectively. In agreement with previous studies [17,18,19], our data suggested that the LAS may be limited to only a few nucleotides, including the adenine at position -2 [21]. In the present study, the LAS sequence of the leader did contain the GAG motif, while spacers 3 and 4 did not have the GAG motif at the 3′ end (just the adenine at position -2). Similar results were observed in *S. pyogenes* [16]. In that case, while the LAS sequence (conserved between the two species) was critical for the integration into the first repeat, one of the spacers may have led the addition of new spacers into its downstream repeat while not fully matching the GAG motif of the leader sequence (only the G at the position -3). Therefore, it could be possible that other factors guide ectopic spacer acquisition. Further experimental studies are needed to address the above.

Nevertheless, these results represent the first study showing ectopic spacer acquisition in the CR3 of *S. thermophilus*. Our analyses also suggest that a similar phenomenon may be naturally occurring in *S. pyogenes,* supporting the previously observed ectopic spacer acquisition in this type II-A system when expressed in *S. aureus* [33]. It should be noted that the acquisition of novel spacers at the 5′ end of the CRISPR array is still the preferred location of new immunities. However, ectopic spacer acquisition also occurs at various frequencies. While CRISPR arrays still represent molecular archives of past nucleic acids invasion, the chronology of these events may not always be correlated with the spacer position within a given array.

## Figures and Tables

**Table 1:** Nucleotide sequence and position of newly acquired ectopic spacers in CRISPR3 of the BIMs obtained after a challenge with phage 53.

BIM	Sequence of New Spacer (5′-3′)	Position in CRISPR3	Protospacer Position in Phage 53 Genome	PAM NGGNG
BIM1	TGATAGTAAAATATTGTCATCATTGAATAC	6	None *	None
	CATTACAGACACAGGAGAAGGCGGCTATTA	4	22837-22866, *orf22*	TGGTG
BIM2	TTATGCAAACGGTGGCCTAGTCCACAAGAA	4	14360-14389, *orf14*	CGGCG
BIM3	AGTTGATGGTAAAACGGTGGAATGACCATA	4	31076-31105, *orf34*	TGGCG
BIM4	AAACGTCAAAAAAGCTGGTAGTAAGGTCAA	4	11789-11818, *orf14*	TGGCG
BIM5	TGTTCAGTATCGTCGACTTCATTCCCCAAA	4	10537-10508, *orf14*	CGGCG
BIM6	TGTTCAGTATCGTCGACTTCATTCCCCAAA	4	10537-10508, *orf14*	CGGCG

* Did not match any region in phage 53 genome and no significant results were obtained from BLAST analysis.

## Data Availability

The data presented in this study are available in the article’s figures and tables and in the Supplementary Materials. The analyzed genome sequences are available on GenBank (NCBI). Additional information are available on request from the corresponding author.

## Supplementary Materials

The following are available online at https://www.mdpi.com/2076-2607/9/3/512/s1, Table S1: Nucleotide sequences and positions of newly acquired spacers in CRISPR3 of all the BIMs obtained after a challenge with the virulent *cos*-type phage 53.
