# Supplementary material for: Ectopic Spacer Acquisition in Streptococcus thermophilus CRISPR3 Array
Source: Microorganisms. 2021 Mar 1;9(3):512. doi: 10.3390/microorganisms9030512 (PMC7999890; doi:10.3390/microorganisms9030512)
Supplement: Supplementary file 1 [file microorganisms-09-00512-s001.pdf]

Table S1. Nucleotide sequences and positions of newly acquired spacers in CRISPR3 of all the BIMs obtained after a challenge with the virulent *cos*-type phage 53.

BIM*	Sequence of new spacer (5′-3′)	Position in CRISPR3	Protospacer position in phage 53 genome	PAM NGGNG
BIM1	TGATAGTAAAATATTGTCATCATTGAATAC	None	None	None
	CATTACAGACACAGGAGAAGGCGGCTATTA	4	22837-22866, *orf22*	TGGTG
BIM2	TTATGCAAACGGTGGCCTAGTCCACAAGAA	4	14360-14389, *orf14*	CGGCG
BIM3	AGTTGATGGTAAAACGGTGGAATGACCATA	4	31076-31105, *orf35*	TGGCG
BIM4-1	AAACGTCAAAAAAGCTGGTAGTAAGGTCAA	4	11789-11818, *orf14*	TGGCG
BIM4-2	AAACGTCAAAAAAGCTGGTAGTAAGGTCAA	4	11789-11818, *orf14*	TGGCG
BIM5-1	TGTTCAGTATCGTCGACTTCATTCCCCAAA	4	10537-10508, *orf14*	CGGCG
BIM5-2	TTGTTCAGTATCGTCGACTTCATTCCCCAAA	4	10538-10508, *orf14*	CGGCG
BIM6	TGTTCAGTATCGTCGACTTCATTCCCCAAA	4	10537-10508, *orf14*	CGGCG
BIM7	TCATTCGTATCAGCTATATGGATGATGTAA	9	1880-1909, *orf2*	AGGAG
BIM8	TGAAGAAATTACGCTTAATATTGCTTCAAA	9	5263-5292, *orf6*	CGGCG
BIM9-1	GAAGGCTCAAGAAAGTCAGATGCGAAGCGC	9	9884-9913, *orf14*	TGGCG
BIM9-2	GAAGGCTCAAGAAAGTCAGATGCGAAGCGC	9	9884-9913, *orf14*	TGGCG
BIM10	TCGTGGAGAAGACGCAGTAAATAATAAACT	9	11846-11875, *orf14*	TGGTG
BIM11-1	CTACCGCTTCAAAGATAAAGCGAAAGACTC	9	13499-13528, *orf14*	TGGAG
BIM11-2	CTACCGCTTCAAAGATAAAGCGAAAGACTC	9	13499-13528, *orf14*	TGGAG
BIM11-3	CTACCGCTTCAAAGATAAAGCGAAAGACTC	9	13499-13528, *orf14*	TGGAG
BIM12	TACTTATGATGGTGAAGATTACAACTATAA	9	13769-13789, *orf14*	CGGTG
BIM13	TAAGATTTTGGCGACTATACAGAAGGAATC	9	14108-14137, *orf14*	AGGCG
BIM14-1	CTCATATTCGTTTGTGGCTTTTGTAATAAA	9	14708-14679, *orf14*	TGGTG
BIM14-2	CTCATATTCGTTTGTGGCTTTTGTAATAAA	9	14708-14679, *orf14*	TGGTG
BIM15-1	TAAACGATACTTGAATCTGTTTAACTGATA	9	14942-14913, *orf15*	AGGTG
BIM15-2	TAAACGATACTTGAATCTGTTTAACTGATA	9	14942-14913, *orf15*	AGGTG
BIM16-1	ATGCGGTTTGATGATGTCGTTCAGTACTAC	9	15896-15925, *orf15*	TGGTG
BIM16-2	ATGCGGTTTGATGATGTCGTTCAGTACTAC	9	15896-15925, *orf15*	TGGTG
BIM16-3	ATGCGGTTTGATGATGTCGTTCAGTACTAC	9	15896-15925, *orf15*	TGGTG
BIM17-1	TTTCAACGCTGGTAATTTCATTGGACAAGA	9	16983-17012, *orf16*	TGGCG
BIM17-2	TTTCAACGCTGGTAATTTCATTGGACAAGA	9	16983-17012, *orf16*	TGGCG
BIM18-1	GAAGACAGCACAAACCCAGCAGATTATACA	9	17899-17928, *orf16*	TGGAG
BIM18-2	GAAGACAGCACAAACCCAGCAGATTATACA	9	17899-17928, *orf16*	TGGAG
BIM18-3	GAAGACAGCACAAACCCAGCAGATTATACA	9	17899-17928, *orf16*	TGGAG
BIM19-1	TTTATCTTTTTCAGCGCAGTTTAACGGGTC	9	19167-19196, *orf17*	TGGTG
BIM19-2	TTTATCTTTTTCAGCGCAGTTTAACGGGTC	9	19167-19196, *orf17*	TGGTG
BIM19-3	TTTATCTTTTTCAGCGCAGTTTAACGGGTC	9	19167-19196, *orf17*	TGGTG
BIM20-1	TGAGCAGGTAGTCAATACATTCAAAAGAAA	9	26313-26342, *orf31*	TGGCG
BIM20-2	TGAGCAGGTAGTCAATACATTCAAAAGAAA	9	26313-26342, *orf31*	TGGCG
BIM21-1	TACATTCTTTGTCATTGACGTGGACATGCA	9	28097-28126, *orf33*	TGGCG
BIM21-2	TACATTCTTTGTCATTGACGTGGACATGCA	9	28097-28126, *orf33*	TGGCG
BIM21-3	TACATTCTTTGTCATTGACGTGGACATGCA	9	28097-28126, *orf33*	TGGCG
BIM22-1	CCACACCCTCCGAATGTCGTTTTCAGTCAT	9	28058-28029, *orf35*	TGGTG
BIM22-2	CCACACCCTCCGAATGTCGTTTTCAGTCAT	9	28058-28029, *orf35*	TGGTG
BIM23	AAGAGGTGCTTCTGTTATGCTTCCATCTTT	9	28406-28377, *orf33*	TGGCG
BIM24	TCAGAATGGCTGATGGACGTTGGTTTGATAC	9	30865-30895, *orf35*	TGGAG
BIM25-1	AGTTGATGGTAAAACGGTGGAATGACCATA	9	31076-31105, *orf35*	TGGCG

BIM25-2	AGTTGATGGTAAAACGGTGGAATGACCATA	9	31076-31105, *orf35*	TGGCG
BIM25-3	AGTTGATGGTAAAACGGTGGAATGACCATA	9	31076-31105, *orf35*	TGGCG
BIM25-4	AGTTGATGGTAAAACGGTGGAATGACCATA	9	31076-31105, *orf35*	TGGCG

*Different BIMs that went through the same acquisition and deletion events were considered the same, therefore named with the same number followed by a hyphen and the replica number. Of note, spacers sequences from BIMs 5-1 and 5-2 differ in one nucleotide. BIM3 and BIMs 25, and BIM5-1 and BIM6 share the same spacer sequence but went through different acquisition and deletion events so were considered different.
